# Rates of bacterial co-infections and antimicrobial use in COVID-19 patients: a retrospective cohort study in light of antibiotic stewardship

**DOI:** 10.1007/s10096-020-04063-8

**Published:** 2020-11-02

**Authors:** Kathrin Rothe, Susanne Feihl, Jochen Schneider, Fabian Wallnöfer, Milena Wurst, Marina Lukas, Matthias Treiber, Tobias Lahmer, Markus Heim, Michael Dommasch, Birgit Waschulzik, Alexander Zink, Christiane Querbach, Dirk H. Busch, Roland M. Schmid, Gerhard Schneider, Christoph D. Spinner

**Affiliations:** 1 Institute for Medical Microbiology, Immunology and Hygiene, Technical University of Munich, School of Medicine, Trogerstr. 30, 81675 Munich, Germany; 2 Department of Internal Medicine II, Technical University of Munich, School of Medicine, Munich, Germany; 3 Department of Anaesthesiology and Intensive Care Medicine, Technical University of Munich, School of Medicine, Munich, Germany; 4 Department of Internal Medicine I, Technical University of Munich, School of Medicine, Munich, Germany; 5 Institute of Medical Informatics, Statistics and Epidemiology, Technical University of Munich, School of Medicine, Munich, Germany; 6 Department of Dermatology and Allergology, Technical University of Munich, School of Medicine, Munich, Germany; 7 Hospital Pharmacy, Technical University of Munich, School of Medicine, Munich, Germany; 8 German Center for Infection Research (DZIF), Partner Site Munich, Munich, Germany

**Keywords:** COVID-19, Antibiotic stewardship, Bacterial co-infections, Diagnostic stewardship

## Abstract

The coronavirus disease 2019 (COVID-19) caused by the severe acute respiratory syndrome coronavirus 2 (SARS-CoV-2) has spread worldwide. Bacterial co-infections are associated with unfavourable outcomes in respiratory viral infections; however, microbiological and antibiotic data related to COVID-19 are sparse. Adequate use of antibiotics in line with antibiotic stewardship (ABS) principles is warranted during the pandemic. We performed a retrospective study of clinical and microbiological characteristics of 140 COVID-19 patients admitted between February and April 2020 to a German University hospital, with a focus on bacterial co-infections and antimicrobial therapy. The final date of follow-up was 6 May 2020. Clinical data of 140 COVID-19 patients were recorded: The median age was 63.5 (range 17–99) years; 64% were males. According to the implemented local ABS guidelines, the most commonly used antibiotic regimen was ampicillin/sulbactam (41.5%) with a median duration of 6 (range 1–13) days. Urinary antigen tests for *Legionella pneumophila* and *Streptococcus peumoniae* were negative in all cases. In critically ill patients admitted to intensive care units (*n* = 50), co-infections with *Enterobacterales* (34.0%) and *Aspergillus fumigatus* (18.0%) were detected. Blood cultures collected at admission showed a diagnostic yield of 4.2%. Bacterial and fungal co-infections are rare in COVID-19 patients and are mainly prevalent in critically ill patients. Further studies are needed to assess the impact of antimicrobial therapy on therapeutic outcome in COVID-19 patients to prevent antimicrobial overuse. ABS guidelines could help in optimising the management of COVID-19. Investigation of microbial patterns of infectious complications in critically ill COVID-19 patients is also required.

**Supplementary Information:**

The online version of this article (10.1007/s10096-020-04063-8) contains supplementary material, which is available to authorized users.

## Introduction

In December 2019, patients presenting with respiratory tract infections due to a formerly unidentified microbial agent were reported in Wuhan, China. A novel beta-coronavirus, severe acute respiratory syndrome coronavirus 2 (SARS-CoV-2), was subsequently identified as the causative pathogen. The corresponding disease was then named coronavirus disease 2019 (COVID-19) by the World Health Organization (WHO) [1, 2]. SARS-CoV-2 is reported to be more infectious than SARS-CoV, and the outbreak led to a pandemic. As of 5 May 2020, more than 3 million cases and over 200,000 deaths due to COVID-19 have been confirmed worldwide. Although most people develop mild or uncomplicated illness, severe disease requiring hospitalisation is also observed in a subset of patients [3]. In severe cases, oxygen support and intensive care unit (ICU) admission are required, and complications such as acute respiratory distress syndrome (ARDS), sepsis, and multi-organ failure are observed [4, 5]. The WHO guidelines for the clinical management of COVID-19 advise clinicians to collect blood cultures (BCs) as well as respiratory samples from the upper respiratory tract for bacterial cultures, and to start empirical antimicrobial treatment only in severe cases [4].

Although the symptoms, clinical course, and risk factors for disease severity associated with COVID-19 have been analysed [6], data on bacterial or fungal co-infections in COVID-19 are sparse. Generally, respiratory viral infections are a risk factor for bacterial co-infections, which then increase disease severity and mortality. It has been shown that sepsis and ventilator-associated pneumonia are a frequently observed complication in COVID-19 patients [7].

In COVID-19 patients, bacterial and fungal co-infection rates are generally low, with higher rates in critically ill ICU-patients, but antimicrobial coverage was noted in the majority of patients [8–10]. Microbiological diagnosis of co-infection is complex and data on pathogens causing such infections in COVID-19 patients are limited, as most studies do not report or identify co-infections in COVID-19 cases [7, 9, 11, 12]. In published case series, bacterial and fungal co-infections in COVID-19 patients due to gram-negative pathogens and *Aspergillus* spp. were described [12–14]. Lack of data on bacterial and fungal cultures may be due to the absence of routine microbiological workup, as health care workers collecting respiratory samples and laboratory technicians processing these samples are at risk of exposure [15]. Notably, the rate of antibiotic use was considerably higher than the incidence of confirmed secondary infections in COVID-19 patients [16]. Based on only a few studies without defined information on sampling strategies, a bacterial or fungal co-infection rate of 8% in COVID-19 patients was estimated, but 72% of all these reported COVID-19 patients received (empiric broad-spectrum) antibiotic therapy [9]. Overuse of antimicrobials increases the risk for multi-resistant nosocomial secondary infections, which are associated with unfavourable clinical outcomes [17]. Therefore, practice of empirical antibiotic coverage in COVID-19 patients must be carefully evaluated.

The goal of antibiotic stewardship (ABS) programmes is to optimise the use of antimicrobial agents while reducing the risks of the emergence of antimicrobial resistance, side effects, and pharmaceutical costs. ABS strategies decrease antimicrobial use; thus, national and international guidelines encourage hospitals to implement local ABS guidelines to optimise treatment and de-escalation strategies [18, 19]. So far, COVID-19-specific ABS interventions are sparse while they have huge beneficial potential in the management of the pandemic by optimising treatment strategies, antibiotic use, and diagnostic stewardship [20–22] . Our institution implemented a local ABS standard operation procedure (SOP) based on Zhang and colleagues [23] on 18 March 2020, for diagnostic measures and empirical antimicrobial coverage with a narrow-spectrum aminopenicillin/beta-lactamase inhibitor combination. Physicians were instructed to collect two sets of BCs and perform a urinary antigen test for *Legionella pneumophila* and *Streptococcus pneumoniae* in each patient with suspected or confirmed COVID-19 upon hospital admission. During hospitalisation, respiratory samples were routinely collected only from critically ill patients.

The present study aimed to analyse the microbiological findings and antibiotic therapies applied in our cohort to widen existing knowledge on bacterial co-infections as well as on antibiotic regimens for COVID-19.

## Materials and methods

We performed a retrospective single-centre cohort study of 140 hospitalised adult patients (age 17–99 years) with confirmed COVID-19, admitted between February 16, 2020, and April 22, 2020, to a large university hospital with approximately 1200 beds, located in the centre of Munich, Germany. Patients were followed up until the 6 May 2020. Medical records including clinical charts and nursing records were reviewed; only the in-hospital patient course was analysed; no further outcome data are available. Initial emergency department (ED) care in patients to be hospitalised was also considered in-hospital patient course. Data collection included patient demographics, comorbidities, clinical parameters, laboratory findings, microbiological analysis information on inpatient management, hospital stay, and outcome as well as antibiotic regimen. Data extraction for laboratory-confirmed COVID-19 patients was performed according to published methods [24, 25]: Relevant variables were defined, documented in an abstraction form, and extracted by two medical students from electronical patient files after abstractor training by an infectious diseases specialist and a clinical microbiologist who also monitored performance of data extraction and were available for inquires. Chart reviewers were not blinded and inter-rater agreement was not tested. Missing patient data was accepted for laboratory parameters and antibiotic therapies due to the retrospective nature of the study. The Ethics Committee of the Technical University of Munich approved the protocol of this retrospective study and waived the need to obtain consent for the collection, analysis, and publication of the data (approval no. 260/20S). Laboratory confirmation of SARS-CoV-2 was achieved by RT-PCR or serological testing. RNA was extracted from swabs using the MagNA Pure 96 system (Roche, Penzberg, Germany). A 2-step in-house RT-PCR protocol as described by Corman et al. [26] was used, including the E gene assay as a screening test, followed by the Rd. gene assay test for confirmation. Serum IgM and IgG antibodies against SARS-CoV-2 were tested using a paramagnetic particle chemiluminescent immunoassay (CLIA) on iFlash immunoassay analyser (Shenzhen Yhlo Biotech Co., Shenzhen, China). Additionally, a chest computed tomography (CT) scan was performed in all patients and evaluated to identify typical CT findings for COVID-19 [27]. Patients with positive PCR or positive IgM/IgG-serology results and symptoms with or without specific COVID-19 CT features were defined as confirmed COVID-19 cases. Suspected but undiagnosed patients were excluded from the analysis.Patients were treated at the ED of our institution according to a local ABS SOP (Supplementary Fig. 1) started on March 18, 2020, and based on Zhang and colleagues [23]. This ABS guideline includes a diagnostic algorithm based on clinical, laboratory, and chest CT findings and advises on the use of microbiological and virological diagnostics as well as empirical antibiotic therapy. The SOP advised on initiation of antibiotic therapy only in cases of clinically suspected infection and elevated inflammatory parameters. The decision to start or change antimicrobial therapy was at the discretion of the physicians. Laboratory COVID-19 test results were only available with delay; therefore, initial diagnosis in the emergency setting was based on clinical condition and chest CT scans which were available and transmitted immediately. Biomarkers were available timely in the ED-setting enabling physicians to base antibiotic therapy on laboratory results. In the standard ED-workflow, laboratory test results are available prior to initiation of antibiotic therapy. However, in our retrospective study setting, we cannot control if laboratory parameters were actually noticed prior to the antibiotic prescription in every case.Urine samples were used for lateral flow antigen test detection of *Legionella pneumophila* serogroup 1 and *Streptococcus pneumoniae* (BinaxNOW®, Abott, Chicago, USA).For microbiological workup, blood was inoculated into aerobic and anaerobic BC media (BACTEC™ Plus, Becton Dickinson, Sparks, MD, USA) via an automated BC system (BACTEC™ Fluorescent Series, Becton Dickinson). Culture bottles were incubated for 5–7 days according to the manufacturer’s recommendations. Considering the difficulty in determining the clinical significance of *coagulase-negative staphylococci* (CoNS) in BC, these isolates were reviewed separately based on the number of positive culture sets, the presence of intravascular devices, and patient’s characteristics. Isolates were considered clinically significant (true bacteraemia) if two or more bottles yielded the same CoNS.

Primary microbiological cultures of respiratory samples were performed on Columbia agar, Schaedler agar, and Chocolate agar (prepared culture media, Becton Dickinson, Sparks, MD, USA). Gram stain identification, species identification (Matrix Assisted Laser Desorption Ionisation-Time of Flight Mass Spectrometry, Bruker Daltronics, Leipzig, Germany), and automated antimicrobial susceptibility testing (VITEK®, bioMerieux, Marcy l’Etoile, France) were performed for all relevant pathogens. Anaerobic strains were tested using minimal inhibitory concentration (MIC) test strips (Liofilchem Inc., Waltham, MA, USA).

German national standards were used to define multidrug-resistant gram-negative bacteria [28]. This national classification defines four antibiotic classes with clinical relevance for treatment of gram-negative infections: carbapenems, quinolones, third-and-fourth-generation cephalosporins and ureidopenicillins. Multi-resistance of gram-negative pathogens is classified as resistance to three of these classes (3MRGN); extensive resistance is defined as resistance to all of these classes or detection of carbapenemase (4MRGN) (Supplementary Table 1).

### Statistics

Continuous data is described by median (range) and categorical data by absolute and relative frequencies. Statistical significance of the association between carbapenem therapy and detection of superinfection was determined with a two-tailed Fisher’s exact test using *P* < 0.05 as the level of significance. Statistical analyses were performed using Microsoft Excel 2013 and IBM SPSS Statistics Version 25.0 (IBM Corp, Armonk, NY).

## Results

During the study period, 140 confirmed COVID-19 patients were evaluated. On the final day of follow-up, 27 patients (19%) were still in inpatient care (13 in general wards and 14 in ICU), 18 patients (13%) died (6 died in general wards, and 12 died in ICU), and 95 patients (68%) were discharged (21 with previous ICU stay and 74 with hospital stay in general wards only).

COVID-19 was diagnosed by PCR in 126 patients (90.0%) and based on serological tests in 14 cases (10.0%). Chest CT-scan findings consistent with COVID-19 symptoms were observed in 114 patients (81.4%) in our cohort. CT findings were unspecific in 6 cases, and a chest CT-scan was not performed in another 6 cases.

Patient characteristics, laboratory findings, microbiological workup, and antibiotic use were analysed for the entire cohort. Moreover, the cohort was divided into two subgroups based on clinical outcome: Subgroup one included patients admitted to the general ward only (*n* = 84: moderate cases). The second subgroup included patients admitted to the ICU and all patients who died during hospital stay, regardless if death occurred on ICU or general ward (*n* = 56: severe cases).

The median age of the cohort was 63.5 (17–99) years, and 90 (64.3%) patients were males. In severe cases, the median age and the number of males were higher. At least one underlying comorbidity was present in 75.7%, with arterial hypertension being the most frequent comorbidity; a higher frequency was observed in more severe cases. The median length of hospital stay was associated with severity of disease. The median duration of ICU stay was 11 days. Twenty-two patients (15.7%) were admitted directly to the ICU and 41 patients (73.2% of all ICU patients) required invasive mechanical ventilation.

The mean C-reactive protein (CRP) level, leukocyte count, and procalcitonin (PCT) level on admission were higher in severe COVID-19 patients than in moderate cases. Detailed demographic and laboratory findings are shown in Table 1.

**Table 1:** Demographic characteristics and laboratory findings

	Entire cohort (*n* = 140)	Severe COVID-19 patients (*n* = 56)	Moderate COVID-19 patients (*n* = 84)
Mean age	63.5 (17–99)	68.5 (26–99)	63 (17–95)
Male sex	90 (64.3%)	40 (71.4%)	50 (59.5%)
Comorbidities			
Presence of any comorbidity as listed below*	106 (75.7%)	43 (76.8%)	63 (75.0%)
- Obesity	23 (16.4%)	12 (21.4%)	11 (13.1%)
- Arterial hypertension	68 (48.6%)	30 (53.6%)	38 (45.2%)
- Diabetes	30 (21.4%)	16 (28.6%)	14 (16.7%)
- Coronary heart disease	26 (18.6%)	12 (21.4%)	14 (16.7%)
- Congestive heart failure	12 (8.6%)	5 (8.9%)	7 (8.3%)
- COPD	7 (5.0%)	6 (10.7%)	1 (1.2%)
- Bronchial asthma	15 (10.7%)	2 (3.6%)	13 (15.5%)
- Chronic kidney disease	16 (11.4%)	10 (17.9%)	6 (7.1%)
- Cancer	29 (20.7%)	15 (26.8%)	14 (16.7%)
- HIV	5 (3.6%)	1 (1.8%)	4 (4.8%)
- Any medical immunosuppression	15 (10.7%)	7 (12.5%)	8 (9.5%)
- Chronic liver disease	7 (5.0%)	4 (7.1%)	3 (3.6%)
Duration of hospital stay (days)	12 (1–47)	19 (1–47)	10 (1–46)
Duration of ICU stay (days)		11 (1–38)	
Interval from hospital admission to ICU admission (days)		1.0 (0–23)	
Laboratory findings on admission			
- CRP (mg/dL)	6.1 (0.1–35)	9.9 (0.3–35.0)	4.7 (0.1–26.6)
- Leukocyte (G/L)	6.4 (1.4–26.3)	7.2 (1.6–22.4)	6.1 (1.4–26.3)
- PCT (ng/mL)	0.1 (0.1–18.7)	0.3 (0.1–18.7)	0.1 (0.1–5.9)
Laboratory findings on day of highest CRP value			
- CRP (mg/dL)	14.8 (0.1–50.6)	27.6 (1.5–50.6)	8.8 (0.1–33.5)
- Leukocyte (G/L)	10.2 (1.8–56.6)	16.1 (5.9–56.6)	7.8 (1.8–37.4)
- PCT (ng/mL)	0.2 (0.1–175.5)	1.8 (0.1–175.5)	0.1 (0.1–138.4)

Data is presented as absolute numbers and relative frequencies (*n* (%)) or as median (range). *COVID-19*, coronavirus disease-2019; *ICU*, intensive care unit; *COPD*, chronic obstructive pulmonary disease; *HIV*, human immunodeficiency virus; *CRP*, C-reactive protein; *PCT*, procalcitonin

*As defined by clinicians in admission records

During hospitalisation, all patients were treated in adequate isolation units to prevent the spread of the disease. Information on antibiotic therapy was not available for five patients which were all treated on general wards only. Of the remaining 135 analysed cases, most patients received antimicrobial therapy within 24 h of admission (*n* = 109, 80.7%): in 113 patients antimicrobial therapy was initiated in the ED, and in 22 patients therapy was initiated on ICU (in cases of direct admittance to ICU). Only 19 patients (14.1%) were not administered any antimicrobial therapy during hospitalisation. The antibiotic regimen most commonly used was ampicillin/sulbactam with or without azithromycin (41.5%) with a median duration of 6 (1–13) days, followed by piperacillin/tazobactam with or without azithromycin (19.3%) with a median duration of 10 (3–26) days. Empirical use of ampicillin/sulbactam is in line with local ABS guidelines. However, the SOP did not advise on the use of azithromycin in combination with beta-lactam antibiotics; nevertheless, it was administered in combination to 43 patients (31.9%).

Interestingly, laboratory findings on admission appear to be different between antibiotic regimens, suggesting that high inflammatory markers influenced the physician’s decision on what antimicrobial to start. Detailed information of initial empirical antibiotic therapy including duration of therapy is presented in Table 2. Administration of (broad-spectrum) antibiotic was observed more frequently in patients with high CRP or PCT values: CRP on admission was higher in patients receiving ampicillin/sulbactam than in patients receiving no antimicrobial therapy. In cases of administration of piperacillin/tazobactam, initial laboratory findings for CRP and PCT were increased. Notably, increased PCT on admission seems to be associated with adverse outcome (i.e. death), as in patients with initial median PCT of 0.1 ng/dL, only 5.4% (3/56) died while the percentage increased to 19.2% (5/26) in patients with initial median PCT of 0.25 ng/dL.

**Table 2:** Most commonly used initial empirical antibiotic therapy

	COVID-19 patients with available information on empiric antibiotic therapy (*n* = 135)	Severe COVID-19 patients with available information on empiric antibiotic therapy (*n* = 56)	Moderate COVID-19 patients with available information on empiric antibiotic therapy (*n* = 79)	Laboratory findings on admission in selected patients
No antibiotic therapy during hospital stay at all	19 (14.1%)	3 (5.4%)	16 (20.3%)	
No initial antibiotic therapy	26 (19.3%)	8 (14.3%)	18 (22.8%)	Leukocyte count 5.7 (3.4–12.5) G/L *CRP 3.6 (0.1–21.4) mg/dL* PCT 0.1 (0.1–2.1) ng/mL
Ampicillin/sulbactam	41 (30.4%)	8 (14.3%)	33 (41.8%)	Leukocyte count 5.6 (1.6–23.1) G/L *CRP 4.8 (0.1–26.6) mg/dL* PCT 0.1 (0.1–15.1) ng/dL
Duration of therapy (days)	7 (1–13)	6 (3–9)	7 (1–13)	
Ampicillin/sulbactam + azithromycin*	15 (11.1%)	9 (16.1%)	6 (7.6%)	
Duration of therapy	4 (1–10)	6 (4–10)	4 (1–7)	
Piperacillin/tazobactam	10 (7.4%)	5 (8.9%)	5 (6.3%)	Leukocyte count 6.8 (2.8–22.6) G/L *CRP 9.3 (0.2–26) mg/dL* *PCT 0.3 (0.1–18.7) ng/mL*
Duration of therapy (days)	9 (5–20)	9 (6–20)	8 (5–15)	
Piperacillin/tazobactam + azithromycin*	16 (11.9%)	10 (17.9%)	6 (7.6%)	
Duration of therapy (days)	10 (3–26)	11.5 (3–26)	10 (7–17)	
Meropenem	1 (0.7%)	1 (1.8%)	0	Leukocyte count 10.3 (4.7–14.5) G/L CRP 9.6 (5.7–17.8) mg/dL PCT 0.1 (0.1–0.8) ng/mL
Duration of therapy (days)	10	10		
Meropenem + azithromycin*	5 (3.7%)	2 (3.6%)	3 (3.8%)	
Duration of therapy (days)	10 (5–25)	18.5 (12–25)	7.5 (5–10)	
Moxifloxacin	4 (3.0%)	2 (3.6%)	2 (2.5%)	Leukocyte count 8.2 (6.3–11.1) G/L CRP 9.0 (6.1–18.1) mg/dL PCT 0.1 (0.1–0.2) ng/mL
Duration of therapy (days)	4 (2–8)	5 (2–8)	4 (3–5)	
Azithromycin	2 (1.5%)	0	2 (2.5%)	Leukocyte count 2.6 (1.4–3.7) G/L CRP 2.9 (1.2–4.5) mg/dL PCT 0.1 (0.1–0.1) ng/mL
Duration of therapy (days)	3	-	3	
Cephalosporin (cefuroxime, ceftazidime, ceftriaxone)	3 (2.2%)	2 (3.6%)	1 (1.3%)	Leukocyte count 7.9 (7.5–8.8.) G/L CRP 8.2 (5.2–35) mg/dL PCT 0.2 (0.2–0.3 ng/mL
Duration of therapy (days)	7 (3–20)	13.5 (7–20)	3	
Initial combination therapy of beta-lactam antibiotic (+/− azithromycin) with vancomycin or linezolid	7 (5.2%)	6 (10.7%)	1 (1.3%)	Leukocyte count 8.8 (6.8–14.9) G/L CRP 18.2 (0.8–35.0) mg/dL PCT 0.3 (0.1–4.9) ng/mL
Duration of therapy (days)	14 (4–25)]	10.5 (4–25)]	18	

Initial antibiotic therapy was defined as antibiotic therapy within 24 h of admission. Information on initial empirical antibiotic therapy was not available for 5 patients. Data is presented as absolute numbers and relative frequencies (*n* (%)). Median duration (days) of every antibiotic regimen is stated as median (range)

Laboratory findings on admission (defined as laboratory results obtained in the ED-encounter which lead to admission of the patient) are presented for different antibiotic regimens

*Median duration of azithromycin-combination-regimens was calculated for beta-lactam antibiotic only; azithromycin was always administered for 3 days. *COVID-19*, coronavirus disease-2019; *ICU*, intensive care unit; *CRP*, C-reactive protein; *PCT*, procalcitonin; *ED*, emergency department

Changes in antibiotic therapy during hospitalisation occurred in 57 COVID-19 patients; of those, 14 patients were moderate cases and 43 patients were severe cases. Supplementary Table 2 displays changes in antibiotic therapy. Details on antimicrobial changes are calculated in reference to these 57 patients. Antibiotic de-escalation in line with ABS principles was not documented in our cohort, but in 11 patients, therapy was discontinued within 3 days of hospital admission as a result of critical revaluation. Escalation was defined as change to broad-spectrum ureidopenicillins and carbapenems with or without glycopeptides/oxazolidinones. Moreover, addition of antifungal therapy was analysed. In a few cases, changes consisted of addition of other antimicrobials such as azithromycin, ceftazidime, ceftriaxone, cefepime, tobramycin, or tigecycline. In 17 patients (29.8%) escalation consisted of piperacillin/tazobactam therapy and theses cases were more frequent among moderate cases (*n* = 9, 64.3%) than in severe cases (*n* = 8, 18.6%). Escalation to meropenem was documented in 9 (15.8%) COVID-19 patients in our cohort with almost equal numbers of more severe cases and moderate cases. Interestingly, escalation to piperacillin/tazobactam plus vancomycin or linezolid was rarely seen (*n* = 1, 1.8%) while the majority of escalations to combination-regimens consisted of meropenem + vancomycin or linezolid (*n* = 20, 35.1%) affecting especially more severe cases (*n* = 19, 44.2%).

Intensification with antimycotic therapy was only observed in more severe cases, echinocandins were added in 5 cases (8.8%), voriconazole in 4 cases (7.0%), fluconazole in 6 cases (10.5%), and addition of liposomal amphotericin-B was documented in 8 (14.0%) COVID-19 patients.

Most patients received microbiological workup upon hospital admission according to the local SOP. In total, BCs were collected from 118 patients (84.3%) and BCs were positive in 10 cases (7.1%). True bacteraemia with confirmed bloodstream infection was detected in only 5 cases, resulting in a BC diagnostic yield of 4.2%. Pathogens considered contaminants were the only cause of bacteraemia in 50% of all positive BCs. Notably, PCT on admission in cases of true bacteraemia was higher than in sterile BCs or contamination only highlighting the diagnostic value of PCT in diagnosing bloodstream infections.

For 57 COVID-19 patients (40.7%), especially in more severe cases, follow-up BCs were collected and relevant pathogens were detected in 11 cases (7.9%).

Urinary antigen tests for *Legionella pneumophila* and *Streptococcus pneumoniae* were performed for 111 (79.3%) and 107 (76.4%) patients, and the results were negative in all cases.

Respiratory samples for microbiological tests were obtained only from a subset of patients on admission (17.9%), all of whom had severe COVID-19 illness. However, the majority of cultures remained sterile or showed the presence of normal oral flora. Relevant pathogens were detected only in 3 cases, where growth of *Escherichia coli* (*n* = 1), *Staphylococcus aureus* (*n* = 1), and *Klebsiella oxytoca* (*n* = 1) was observed.

Most respiratory samples for follow-up microbiological tests were collected from patients admitted to the ICU (*n* = 50). Only one respiratory sample was collected from a patient not admitted to the ICU, and it showed the presence of *Klebsiella aerogenes*. For ICU patients, respiratory samples were collected from 38 (76.0%) patients for follow-up microbiological tests, and relevant pathogens were detected in 23 cases (46.0%), showing a predominance of *Enterobacterales* and *Aspergillus fumigatus.* The pathogens detected in the samples were *Enterobacter cloacae* (*n* = 1), *Serratia marcescen*s (*n* = 4), *Proteus* spp. (*n* = 3), *E. coli* (*n* = 6), *Morganella morganii* (*n* = 1), *Citrobacter* spp. (*n* = 3), *Klebsiella* spp. (*n* = 11), *A. fumigatus* (*n* = 9), *S. aureus* (*n* = 3), and *P. aeruginosa* (*n* = 2).

Neither multidrug-resistant gram-negative pathogen according to the German national classification nor a methicillin-resistant *Staphylococcus aureus* (MRSA) was detected in the cohort. Interestingly, two blood infections with vancomycin-resistant *E. faecium* were detected, with molecular confirmation of VanB in both cases. Table 3 summarises the results of the microbiological tests conducted in the present study.

**Table 3:** Results of microbiologic diagnostics on admission and further relevant microbiological findings during hospitalisation

	Full COVID-19 cohort (*n* = 140)	Severe COVID-19 patients (*n* = 56)	Moderate COVID-19 patients (*n* = 84)
BC collected	118 (84.3%)	52 (92.9%)	66 (78.6%)
BC positive	10 (7.1)	5 (8.9%)	5 (6.0%)
Contamination only	5 (3.6%)	1 (1.8%)	4 (4.8%)
BC pathogen in confirmed, true bacteraemia		*E. coli* (*n* = 1), *S. aureus* (*n* = 1), *S. epidermidis* (*n* = 2)	*E. coli* (*n* = 1)
PCT on admission in true bacteraemia	5.3 (0.8–18.7)	5.55 (0.8–18.5)	5.9
PCT on admission in sterile BC or contamination only	0.1 (0.1–15.1)	0.2 (0.1–15.1)	0.1 (0.1–4.5)
Follow-up BC diagnostic	57 (40.7%)	45 (80.4%)	12 (14.3%)
Cases with positive follow-up BC	11 (7.9%)	10 (17.9%)	1 (1.2%)
Pathogens in positive follow-up BC		*K. oxytoca* (*n* = 1), *K. pneumoniae* (*n* = 1), *P. aeruginosa* (*n* = 1), *S. epidermidis* (*n* = 2), *C. albicans* (*n* = 1), VRE (*n* = 1), VRE + *C. albicans* (*n* = 1), *S. epidermidis* + *C. albicans* (*n* = 1), *S. epidermidis* + *E. faecium* + *K. varicola* (*n* = 1)	*S. aureus* (*n* = 1)
*L. pneumophila* antigen test performed; positive rates	111 (79.3%); 0	47 (83.9%); 0	64 (76.2%); 0
*S. pneumoniae* urinary test performed; positive rates	107 (76.4%); 0	43 (76.8%); 0	64 (76.2%); 0
Respiratory samples collected at admission	25 (17.9%)	25 (44.6%)	0
Clinically relevant pathogens in respiratory samples at admission*	3 (2.1%)	3 (5.4%) *E. coli* (*n* = 1), *S. aureus* (*n* = 1), *K. oxytoca* (*n* = 1)	0
Follow-up microbiological workup of respiratory samples in ICU patients (*n* = 50)^+^		Follow-up samples collected: 38 (76.0%) Detection of relevant pathogens: 23 (46.0%) Polymicrobial infection: 13 (26.0%) Findings per patient *Enterobacterales*: 17 (34.0%) *A. fumigatus*: 9 (18.0%) *S. aureus*: 3 (6.0%) *P. aeruginosa*: 2 (4.0%)	

Data is presented as absolute numbers and relative frequencies (*n* (%))

*Respiratory samples at admission were mainly sputum cultures. Sterile cultures and detection of normal oral flora were regarded as negative in contrast to the detection of relevant pathogens

^+^Relevant number of follow-up microbiological tests of respiratory samples was only available for ICU patients

*COVID-19*, coronavirus disease-2019; *ICU*, intensive care unit; *S. pneumoniae*, *Streptococcus pneumoniae*; *L. pneumophila*, *Legionella pneumophila*; E. *coli*, *Escherichia coli*; *S. aureus*, *Staphylococcus aureus*; *K*. *pneumoniae*, *Klebsiella pneumoniae*; *C*. *albicans*, *Candida albicans*; *P*. *aeruginosa*, *Pseudomonas aeruginosa*; *E. faecium*, *Enterococcus faecium*; *A. fumigatus*, *Aspergillus fumigatus*; *VRE*, vancomycin-resistant enterococci; *MRSA*, methicillin-resistant *S. aureus*; *BC*, blood culture, *PCT*, procalcitonin

To further investigate the predominance of *Enterobacterales* and *Aspergillus fumigatus* in respiratory samples of ICU patients receiving broad-spectrum antimicrobial therapy, the patients were divided into two groups: patients receiving carbapenem therapy and those not receiving carbapenem therapy. Primary or escalation therapy was considered, because retrospective time correlation between the onset of therapy and the microbiologic findings was not possible for all cases. The association between superinfection with *Enterobacterales* or *Aspergillus fumigatus* and carbapenem therapy was evaluated. Among the patients without microbiologically detectable co-infection (*n* = 28, 56.0%), 14 patients (50.0%) did not undergo carbapenem therapy. Among the patients with superinfection with *Enterobacterales*, 6 patients (46.2%) did not undergo carbapenem-based therapy. However, *Aspergillus fumigatus* was detected in the respiratory samples of only 2 patients (22.2%) who had not received carbapenem therapy, possibly indicating the adverse antibiotic effect of a broad-spectrum antimicrobial in terms of increased susceptibility to fungal colonisation (Supplementary Fig. 2). The association of the use of carbapenems with the detection of *Aspergillus fumigatus* was not statistically significant (*p* = 0.427) and has limited validity due to low patient numbers. Also, this finding must be interpreted with caution as use of carbapenems may just represent more severe cases with *Aspergillus fumigatus* as a bystander. Nevertheless, fungal coinfections are an interesting finding in COVID-19 patients [14] and our observation that mainly patients with broad-spectrum-antibiotic therapy are affected is still noteworthy and needs to be addressed in further studies.

## Discussion

This descriptive study on the microbiological testing results of COVID-19 patients showed that, at the time of admission, bacteraemia and *L. pneumophila* or *S. pneumoniae* infection were rarely present, but 46.0% of severely ill patients admitted to the ICU developed bacterial and fungal co-infections, especially due to *Enterobacterales* and *A. fumigatus*.

In patients with clinically suspected bacterial infection or pneumonia, empirical antimicrobial therapy is recommended to be timely initiated even if a causative microorganism cannot be unequivocally identified and often before the results of microbiological diagnostics are available. Antimicrobial treatment might prevent secondary infections and reduce complication rates. However, only pathogen identification and susceptibility testing allows the de-escalation of empirical antimicrobial therapy [29] and increases our knowledge of the bacterial spectrum and antimicrobial resistance, which represents a major pillar of ABS [30]. Diagnostic stewardship is an integral part and the basis for ABS intervention as the appropriate use of microbial diagnostics results in a more sophisticated diagnosis, and can therefore optimise patient management [31, 32]. Multidisciplinary approaches to diagnostic stewardship, as part of an ABS programme, include the development of local guidelines for sample collection. Diagnostic bundles, especially in the ED setting, have been shown to increase the detection of sepsis [33], which is vital for optimal patient care because mortality is reduced when the correct antimicrobial therapy based on the knowledge of local resistance is initiated promptly [34, 35].

Of note, some COVID-19 patients presenting to the ED parallely suffer from additional infectious diseases such as urinary tract infections or skin and soft tissue infections. This must be considered for initiation of adequate diagnostics and empirical therapy. Therefore, requirement of antimicrobial therapy cannot only be only attributed to COVID-19 in our cohort which and must be taken into account when evaluating antibiotic use.

It is of paramount importance for physicians to be aware of the expected spectrum of pathogens of bacterial co-infections in COVID-19 and the corresponding antimicrobial susceptibilities.

Data on bacterial and/or fungal co-infections is sparse, and studies are not consistent regarding the sampling strategies. A 6% rate of bacteraemia, without further specification, at hospital admission has been reported for 393 COVID-19 patients [36]. These results must be interpreted with caution since there might be a high rate of contamination like in our study, perhaps due to bulky personal protective equipment. In line with our findings, in a report on the microbiological testing of respiratory samples from 99 COVID-19 patients, culture (gram-negative and fungal) growth was only observed for 2 cases [9, 12]. Widespread antimicrobial use in COVID-19 patients is common, with quinolones being the most commonly used antimicrobials, followed by carbapenems and cephalosporins [5, 37]. Some national guidelines even specifically advice on the use of broad-spectrum antimicrobials [38].

During the 2003 SARS-CoV-1 outbreak, an increase in MRSA rates among patients admitted to the ICU was noted, and this observation was attributed to the extensive use of broad-spectrum antimicrobials, especially that of cefepime, carbapenems, and fluoroquinolones [39].

ABS in times of COVID-19 is challenging due to biosafety issues for medical staff and laboratory personnel. Guidelines should focus on adequate sampling strategies prior to antibiotic administration and targeted antimicrobial therapy to restrict the overuse of antibiotics to fight emerging resistance and the side effects of antimicrobials. ABS principles could be highly relevant in the management of COVID-19 patients [40]. We implemented local ABS guidelines for diagnostic and therapeutic strategies for suspected COVID-19 patients. Adherence to the guidelines in terms of diagnostics was > 75%, but the detected widespread use of macrolide antibiotics was not favoured by the local ABS guideline. This observation might be due to the overlap of the incidence of COVID-19 with seasonal influenza in spring 2020. Macrolide antibiotics are often initiated to treat atypical pathogens such as *Legionella pneumophila*. These substances show severe side effects [41] which has to be weighed against the apparently low prevalence of atypical organisms in COVID-19 patients [42].

BCs are the gold standard and the most important first-line tool for diagnosing severe bacterial infections [43, 44]; however, the diagnostic value of obtaining BCs in the ED setting has been questioned due to frequent false-positive results from contamination and low positivity rates [45]. In our cohort, the diagnostic yield of BCs seems to be remarkably low, but PCT values were higher in patients with true bacteraemia than in those with sterile BC or contaminated BC. Using PCT as a surrogate marker might help to perform more specific BC diagnostics. Also, PCT was higher in the more severe cases on admission. PCT as a marker for bacterial infections can help to reduce empirical antibiotic use [9, 46]. In our cohort, elevated PCT levels were associated with the use of broad-spectrum antimicrobials, suggesting PCT was used to identify more severe bacterial infections. According to national and international sepsis guidelines, administration of broad-spectrum antimicrobial therapy is recommended in severe infections, as indicated by increased inflammatory markers.

Microbiological test results were available mainly for intubated ICU patients, and co-infections with *Enterobacterales* and *A. fumigatus* were predominantly observed for these patients. Neither multidrug-resistant gram-negative pathogens nor MRSA were detected in the cohort, but two ICU patients presented with VRE bloodstream infection, highlighting the significance of repetitive microbiological testing in COVID-19 patients admitted in the ICU. Nevertheless, (empirical) carbapenem use was high in ICU patients potentially reflecting severity of disease but still rational revaluation of antibiotic therapy is warranted. The exact incidence of bacterial co-infections in COVID-19 is still unknown [40]. Expansion of microbiological testing of respiratory samples for non-ICU patients should be perused, with adequate laboratory staff safety measures, to further increase our knowledge on bacterial co-infections in COVID-19.

The results of the present study should be interpreted with caution due to the limited cohort size. However, considering the number of patients included in the study, we believe that this is a representative COVID-19 cohort for Germany. Larger clinical studies are needed to elucidate the epidemiology, clinical course, and prognostic factors of bacterial co-infections in COVID-19. Due to the nature of the study, only the in-hospital patient course was analysed, no further follow-up data were available, and at the time of analysis, some patients were still hospitalised and undergoing antibiotic treatment, meaning that the final clinical outcome cannot be stated. Due to the retrospective design of this study, respiratory samples were not collected from all patients due to safety concerns. Additional studies on the microbiological findings for COVID-19 patients are needed.

In conclusion, there is a paucity of data on bacterial co-infections in COVID-19 patients. In this study, we found that antimicrobials were being administered at a high rate, but the number of confirmed bacterial infections was low. Severe cases of COVID-19 admitted to the ICU tended to suffer from higher rates of pulmonary enterobacterial and *Aspergillus* infections than patients with less severe forms of the disease. In future studies, the implication of ABS measures and use of antibiotics in COVID-19 should be carefully assessed, considering the low confirmed rates of bacterial infection.

## Electronic supplementary material

Supplementary Figure 1 **Local COVID-19 ABS SOP**. The ABS guideline includes a diagnostic algorithm based on clinical, laboratory and chest CT findings and advises on the use of microbiological and virological diagnostics as well as empirical antibiotic therapy. Abbreviations: COVID-19: Coronavirus disease-2019; ABS: Antibiotic Stewardship; SOP: Standard Operating. Procedure; CT: Computed Tomography (PDF 124 kb)

Supplementary Figure 2 **Association of coinfections with antibiotic use in COIV-19-patients on ICU**. ICU patients were divided into two groups: patients who received meropenem therapy and those who did not receive meropenem therapy (primary or escalation therapy was considered retrospectively as onset of therapy was not available for all cases). Data are presented as absolute numbers and relative frequencies [n (%)]. Cases with no microbiologically detected superinfection are compared to cases with detected superinfection with *Enterobacterales* only and with *Aspergillus fumigatus* ± *Enterobacterales.* Abbreviations: COVID-19: Coronavirus disease-2019; ICU: Intensive Care Unit. (PNG 231 kb)

High Resolution Image (TIF 1453 kb)

ESM 1 (DOCX 15 kb)

## Data Availability

All relevant data are made available in the manuscript and supplementary files.
