# Supplementary material for: Rates of bacterial co-infections and antimicrobial use in COVID-19 patients: a retrospective cohort study in light of antibiotic stewardship
Source: Eur J Clin Microbiol Infect Dis. 2020 Nov 2;40(4):859–69. doi: 10.1007/s10096-020-04063-8 (PMC7605734; doi:10.1007/s10096-020-04063-8)

Klinikum rechts der Isar Technische Universität München

Procedure for patients with suspected COVID-19 at university hospital Klinikum rechts der Isar

Emergency Department algorithm for patients with suspected COVID-19

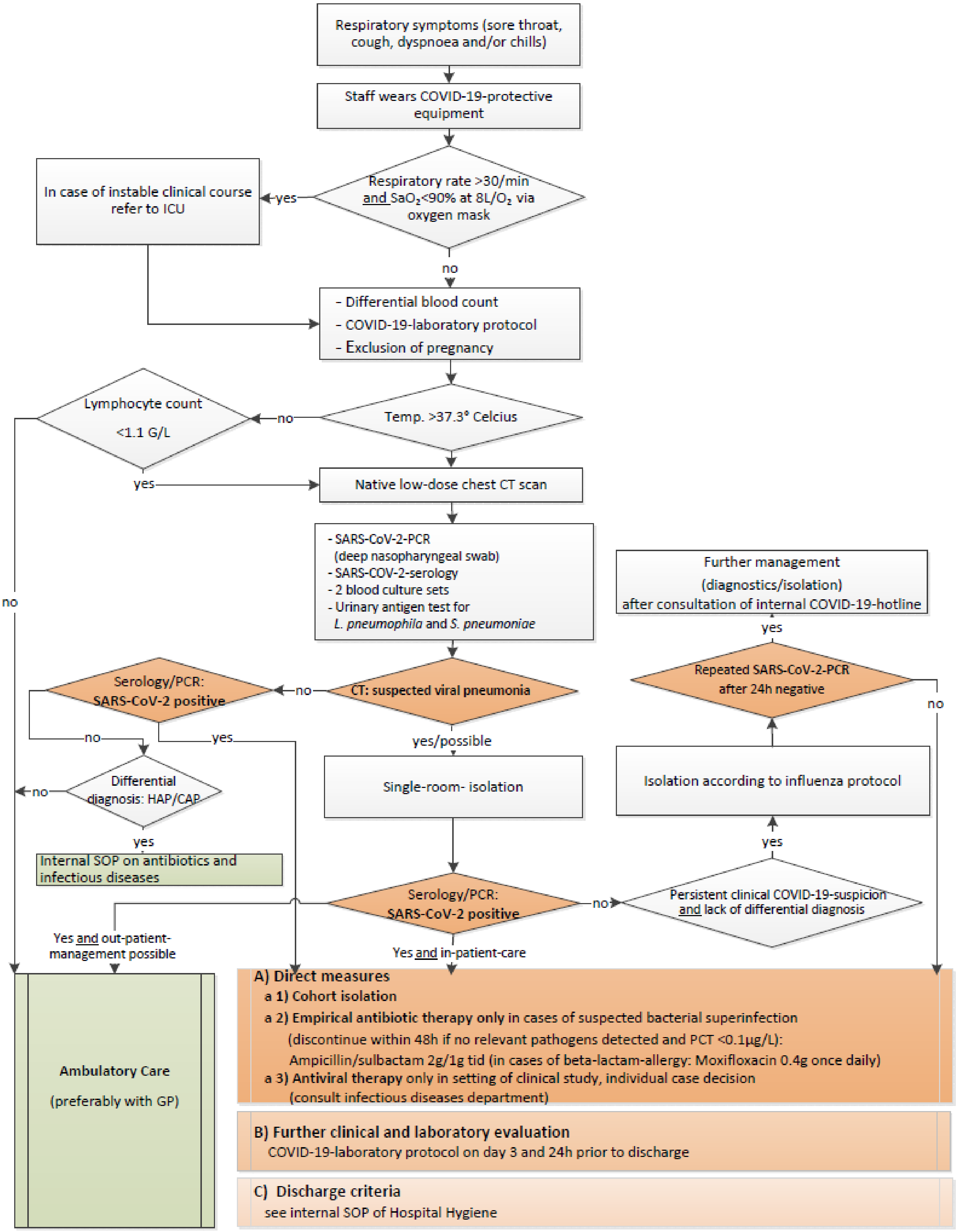

Supplement: Supplementary file 1 — Local COVID-19 ABS SOP. The ABS guideline includes a diagnostic algorithm based on clinical, laboratory and chest CT findings and advises on the use of microbiological and virological diagnostics as well as empirical antibiotic therapy. Abbreviations: COVID-19: Coronavirus disease-2019; ABS: Antibiotic Stewardship; SOP: Standard Operating. Procedure; CT: Computed Tomography (PDF 124 kb) [file 10096_2020_4063_MOESM1_ESM.pdf]
