# Supplementary figures and images for: Rates of bacterial co-infections and antimicrobial use in COVID-19 patients: a retrospective cohort study in light of antibiotic stewardship
Source: Eur J Clin Microbiol Infect Dis. 2020 Nov 2;40(4):859–69. doi: 10.1007/s10096-020-04063-8 (PMC7605734; doi:10.1007/s10096-020-04063-8)

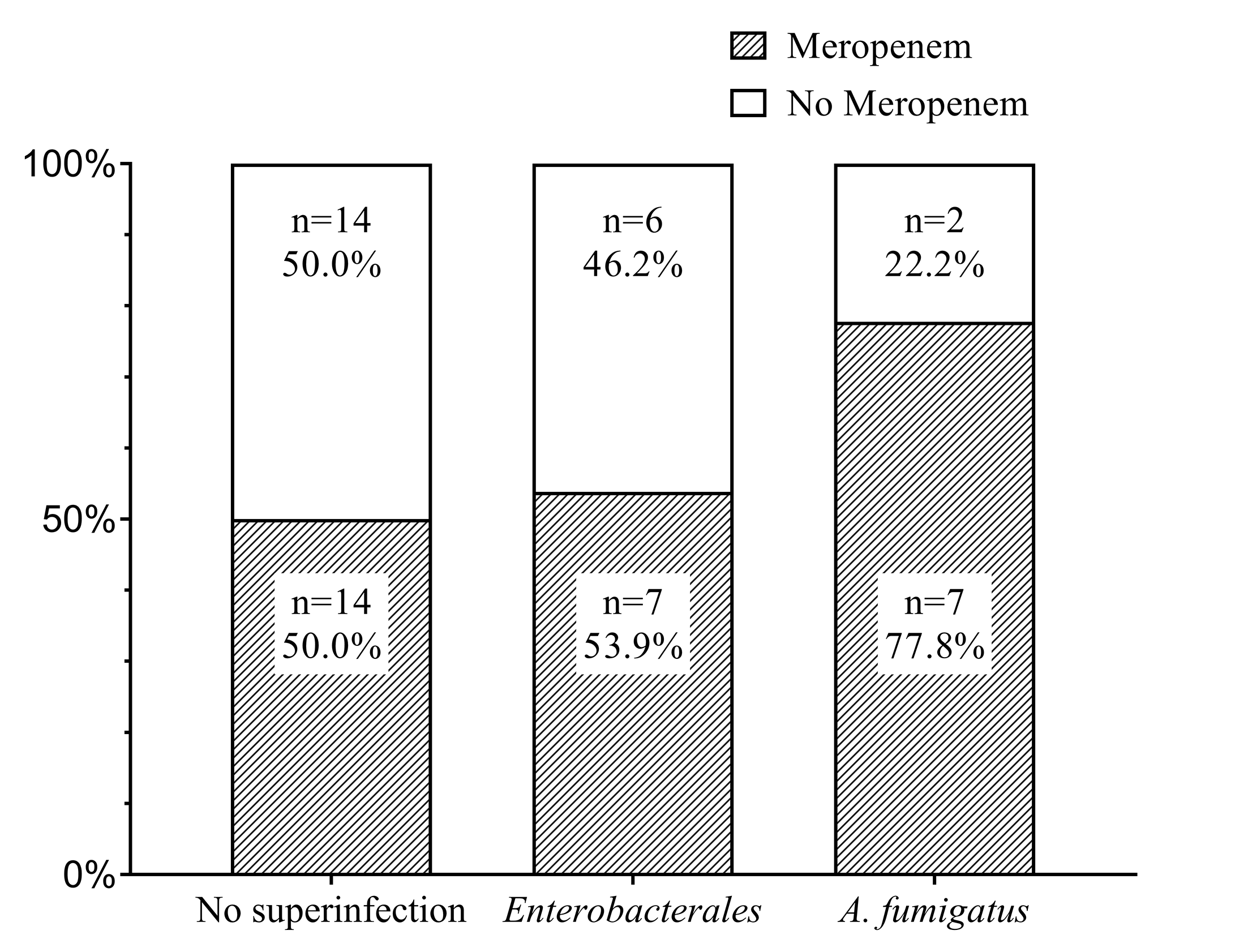

Supplement: Supplementary file 2 — Association of coinfections with antibiotic use in COIV-19-patients on ICU. ICU patients were divided into two groups: patients who received meropenem therapy and those who did not receive meropenem therapy (primary or escalation therapy was considered retrospectively as onset of therapy was not available for all cases). Data are presented as absolute numbers and relative frequencies [n (%)]. Cases with no microbiologically detected superinfection are compared to cases with detected superinfection with Enterobacterales only and with Aspergillus fumigatus ± Enterobacterales. Abbreviations: COVID-19: Coronavirus disease-2019; ICU: Intensive Care Unit. (PNG 231 kb) [file 10096_2020_4063_Fig1_ESM.png]

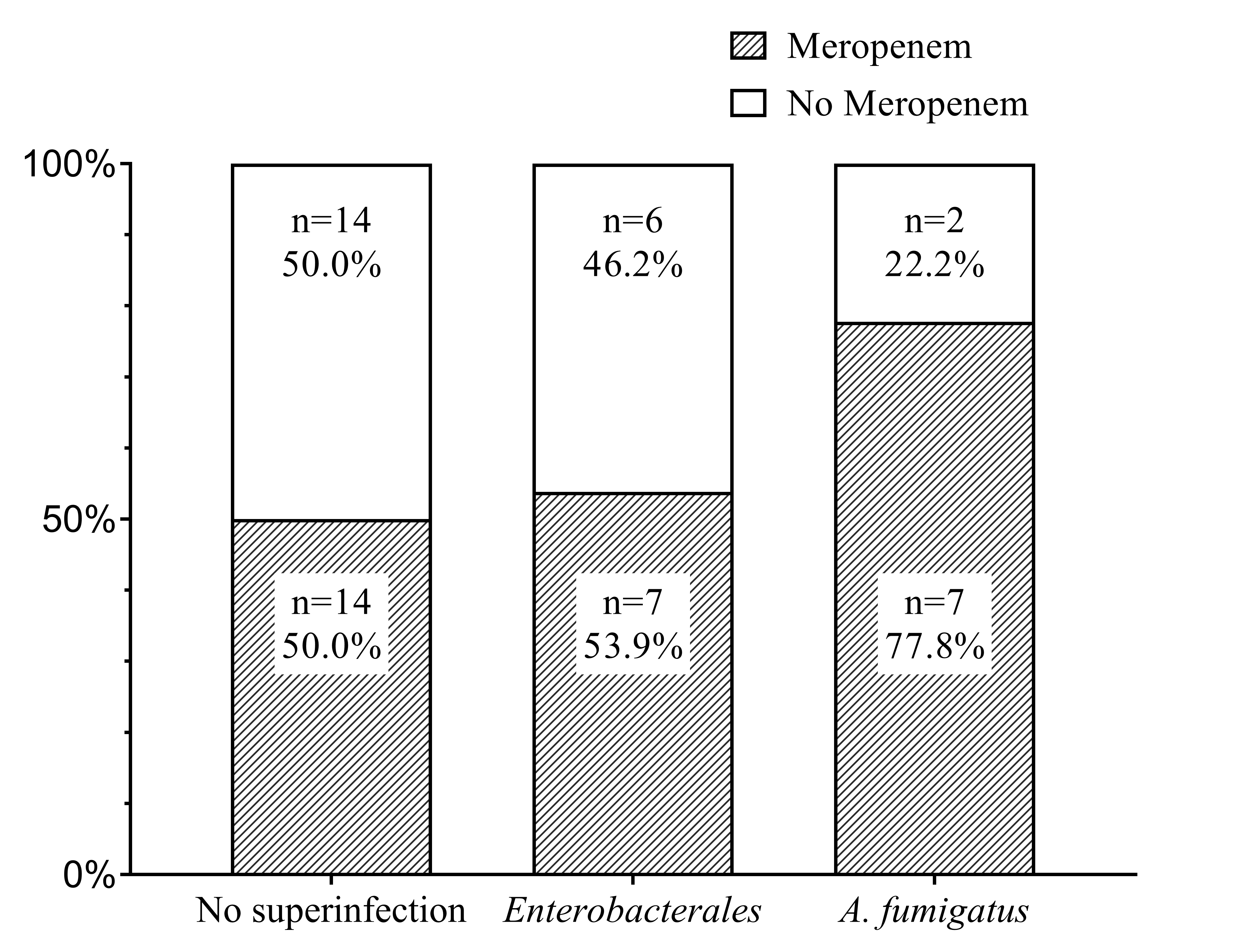

Supplement: Supplementary file 3 — High Resolution Image (TIF 1453 kb) [file 10096_2020_4063_MOESM2_ESM.tif]
