# Supplementary material for: Rates of bacterial co-infections and antimicrobial use in COVID-19 patients: a retrospective cohort study in light of antibiotic stewardship
Source: Eur J Clin Microbiol Infect Dis. 2020 Nov 2;40(4):859–69. doi: 10.1007/s10096-020-04063-8 (PMC7605734; doi:10.1007/s10096-020-04063-8)
Supplement: Supplementary file 4 — (DOCX 15 kb) [file 10096_2020_4063_MOESM3_ESM.docx]

**Supplementary Table 1: Classification of antimicrobial resistance of gram-negative pathogens according to German national guidelines**

| Antimicrobial Categories | | | | Classification | |
| --- | --- | --- | --- | --- | --- |
| I | II | III | IV | 3MRGN | 4MRGN |
| Piperacillin | Cefotaxime OR Ceftazidim | Ciprofloxacin | Imipenem  OR Meropenem OR Carbapenemase detected | Resistance to three out of four categories | Resistance to four out of four categories OR Carbapenemase detected |

Intermediate category of susceptibility testing is considered as susceptible for the classification. Imipenem is not considered for *Proteus spp., Morganella spp., Serratia. marcescens, Providencia spp.* For classification of *Pseudomonas aeruginosa,* resistance to both substances named in category II and IV must be present simultaneously to qualify as resistant

**Supplementary Table 2: Changes in antibiotic therapy**

|  | COVID-19 patients with changes of antibiotic therapy (n=57) | Severe COVID-19- patients with changes of antibiotic therapy (n=43) | Moderate COVID-19- patients with changes of antibiotic therapy (n=14) |
| --- | --- | --- | --- |
| Piperacillin / Tazobactam | 17 (29.8%) | 8 (18.6%) | 9 (64.3%) |
| Piperacillin / Tazobactam + Vancomycin or Linezolid | 1 (1.8%) | 1 (2.3%) | 0 |
| Meropenem | 9 (15.8%) | 7 (16.3%) | 2 (14.3%) |
| Meropenem + Vancomycin or Linezolid | 20 (35.1%) | 19 (44.2%) | 1 (7.3%) |
| Intensification with echinocandin | 5 (8.8%) | 5 (11.6%) | 0 |
| Intensification with Voriconazole | 4 (7.0%) | 4 (9.3%) | 0 |
| Intensification with Fluconazole | 6 (10.5%) | 6 (14.0%) | 0 |
| Intensification with liposomal Amphothericin B | 8 (14.0%) | 8 (18.6%) | 0 |

Changes in primary antibiotic therapy were made in 57 COVID-19 patients. Escalation was defined as change to broad-spectrum ureidopenicillins and carbapenems with or without glycopeptides/ oxalinezolid. In a few cases, changes consisted of addition of other antimicrobials. Moreover, addition of antimycotic therapy was analyzed individually from changes in antibiotic therapy so columns do not necessarily sum up to 100%. Data is presented as absolute numbers and relative frequencies [n (%)].

Abbreviations: COVID-19: Coronavirus disease-2019; ICU: Intensive Care Unit;
